# Defining benchmarks for total and distal gastrectomy: global multicentre analysis

**DOI:** 10.1093/bjs/znad379

**Published:** 2024-02-20

**Authors:** Marcel André Schneider, Jeesun Kim, Felix Berlth, Yutaka Sugita, Peter P Grimminger, Takeshi Sano, Riccardo Rosati, Gian Luca Baiocchi, Maria Bencivenga, Giovanni De Manzoni, Souya Nunobe, Han-Kwang Yang, Christian Alexander Gutschow, GastroBenchmark Consortium, Bas P L Wijnhoven, Hidde Overtoom, Ines Gockel, René Thieme, Ewen A Griffiths, William Butterworth, Henrik Nienhüser, Beat Müller, Nerma Crnovrsanin, Felix Nickel, Suzanne Gisbertz, Mark van Berghe Henegouwen, Philip H Pucher, Kashuf Khan, Asif Chaudry, Pranav H Patel, Manuel Pera, Mariagiulia Dal Cero, Carlos Garcia, Guillermo Martinez Salinas, Paulo Kassab, Osvaldo Antônio Prado Castro, Enrique Norero, Paul Wisniowski, Luke Randall Putnam, Pietro Maria Lombardi, Giovanni Ferrari, Rita Gudaityte, Almantas Maleckas, Leanne Prodehl, Antonio Castaldi, Michel Prudhomme, Simone Giacopuzzi, Francesco Puccetti, Domenico D'Ugo, Daniel Gero, Hyuk-Joon Lee, GASTRODATA Consortium, Guillaume Piessen, Justine Lerooy, Johanna Wilhelmina van Sandick, John V Reynolds, Paolo Morgagni, Arnulf H Hölscher, Martin Hemmerich, Stefan Mönig, Mickael Chevallay, Piotr Kołodziejczyk, Henk Hartgrink, Paulo Matos da Costa, Filipe Castro Borges, Andrew Davies, Cara Baker, William Allum, Sacheen Kumar, Wojciech Polkowski, Karol Rawicz-Pruszyński, Uberto Fumagalli Romario, Stefano De Pascale, Antonio Tarasconi, Daniel Reim, Ilaria Pergolini, Lucio Lara Santos, Pedro Carvalho Martins, Alberto Biondi, Maurizio Degiuli, Rossella Reddavid, Wojciech Kielan, Paul Magnus Schneider, Thomas Murphy

**Affiliations:** Department of Surgery and Transplantation, University Hospital Zurich, Zurich, Switzerland; Department of Surgery, Seoul National University Cancer Hospital, Seoul, South Korea; Department of General, Visceral and Transplant Surgery, University Medical Centre Mainz, Mainz, Germany; Department of Gastroenterological Surgery, Cancer Institute Hospital of the Japanese Foundation for Cancer Research, Tokyo, Japan; Department of General, Visceral and Transplant Surgery, University Medical Centre Mainz, Mainz, Germany; Department of Gastroenterological Surgery, Cancer Institute Hospital of the Japanese Foundation for Cancer Research, Tokyo, Japan; Department of Surgery, San Raffaele Hospital, Milan, Italy; Department of Surgery, University Hospital of Brescia, Brescia, Italy; Department of Surgery, University Hospital of Verona, Verona, Italy; Department of Surgery, University Hospital of Verona, Verona, Italy; Department of Gastroenterological Surgery, Cancer Institute Hospital of the Japanese Foundation for Cancer Research, Tokyo, Japan; Department of Surgery, Seoul National University Cancer Hospital, Seoul, South Korea; Department of Surgery and Transplantation, University Hospital Zurich, Zurich, Switzerland

## Introduction

Gastric cancer is the fifth most common cancer worldwide and ranks third for cancer-related death^1,2^. Curative surgery with or without perioperative chemotherapy remains the therapeutic cornerstone^3,4^; however, oncological gastric resections require particular skills and experience, have a clear correlation between case volume and outcome^5^, and a relevant learning curve^6^.

Oncological gastrectomy is associated with significant postoperative morbidity, with complication and mortality rates ranging from 11% to 46% and 3% to 20% respectively^7–12^. The wide discrepancy in reported outcomes is due to the fact that the current evidence on morbidity after gastrectomy is derived from patients who underwent surgery in centres with variable experience and caseloads. In addition, the current definitions of postoperative morbidity are inconsistent and the documentation quality is variable. Recently, the Gastrectomy Complications Consensus Group (GCCG) curated a list of defined complications after gastrectomy^9^, which was an important step toward standardized outcome reporting. Nevertheless, it can be assumed that the available data provide only a rough guide and do not allow surgeons or centres to compare their own results.

With this in mind, this multicentre benchmark analysis of oncological gastrectomy was conceived. Benchmarking involves identifying a point of reference against which a third party’s performance can be compared. By definition, a benchmark describes a ‘best possible’ outcome under ideal conditions^13^. Benchmarking is a well-established management tool for evaluating efficiency and productivity^14^. With a growing need to monitor outcomes, benchmarking is increasingly used in the field of surgery, especially for complex and cost-intensive procedures^15–24^. In the present study, data were derived from selected ‘optimal’ patients with low co-morbidity, managed by expert institutions on five continents. To account for disease-related and patient-related differences between East Asian and European/American centres, separate benchmark values were calculated per world region. These results can be implemented as a reference owing to the novelty and the relevance of the benchmark concept and the lack of comparable outcome values for oncological gastrectomy in the literature.

## Methods

### Ethics

This study involving data of human participants was performed in accordance with the ethical standards of the respective institutional and/or national research committees and with the 1964 Declaration of Helsinki and its later amendments or comparable ethical standards. Approval from the ethics committees of the leading centre in Zurich in Switzerland (BASEC No. 2022-00931) and each participating centre was obtained before patient inclusion.

### Data collection

Study design and calculation of benchmark values followed a recommended standardized and validated methodology^13,25–27^ and was performed in collaboration with the GASTRODATA group^9,10^.

Planned centre inclusion criteria were an average annual caseload of ≥ 20 oncological gastrectomies, the availability of a prospective database, and a special commitment to upper gastrointestinal surgery as documented by recent publications. Consecutive patients undergoing gastric resections for adenocarcinoma between 1 January 2017 and 31 December 2021 were included in the overall data collection (*Appendix S1*).

De-identified patient-specific data were submitted via secured file transfer and then audited for completeness. In agreement with each participating centre, no data were reported with patient or hospital identifiers. The information collected included basic demographics, ASA and WHO/Eastern Cooperative Oncology Group (ECOG) grades, tumour-specific parameters, technical details of the surgical procedure, and postoperative complications. Endpoints for analysis of postoperative events were at 90 days after surgery. Complications were classified as specified in the databases of the Seoul National University Hospital (South Korea)^28,29^ and the GASTRODATA collaborative (European Chapter of the International Gastric Cancer Association)^9,30^ and were graded according to the Clavien–Dindo (CD) classification^31^. Cumulative morbidity was assessed with the comprehensive complication index (CCI), which expresses morbidity on a continuous scale from 0 (no complications) to 100 (death) by weighing all postoperative complications according to the CD classification^32^.

### Inclusion criteria for low co-morbidity

Benchmark patients were defined as aged 18–65 years, with an ASA grade ≤ II, an ECOG ≤ 1, a BMI between 18 and 30 kg/m^2^, and no significant co-morbidities (see *Appendix S2*). Only patients after total gastrectomy (TG) or distal/subtotal gastrectomy (DG) with adequate lymphadenectomy (D1+, D2, or D2+) from centres that had logged at least 50 cases were eligible for the current benchmark analysis (*Table S1*). Exclusion criteria were histology other than adenocarcinoma, other limited types of gastric resections, concurrent major organ resections, administration of hyperthermic intraperitoneal chemotherapy (HIPEC), relevant prior abdominal surgery, and advanced tumour stage (pT4b, pM1).

### Performance metrics of benchmarking

Primary outcome measures for benchmark analysis of surgical quality were duration of hospital stay, rate of R0 resection, number of lymph nodes resected, rates of blood transfusion, escalation of care, reoperation, readmission, and overall and major (CD grade ≥ IIIA) morbidity, cumulative morbidity as measured by the CCI (all at 30 days), and rates of 30- and 90-day mortality.

According to the benchmarking methodology^13,25–27^, median values of continuous variables and the proportions of categorical variables were calculated per participating centre. Benchmark cut-offs, indicating ‘best achievable’ results for each outcome indicator, were set at the 25th percentile for positive parameters (for example lymph node yield) or the 75th percentile for negative parameters (for example complications) of the centres’ median values.

### Statistical analysis

Statistical significance was defined as *P* < 0.050. Categorical variables are presented as number or % and were compared using Fisher’s exact test and numerical variables are expressed as median and interquartile range (i.q.r.) and were compared using Wilcoxon’s rank sum test. Predictive factors were identified by multivariable logistic regression with respective ORs with 95% confidence intervals. *R* version 4.0.2 (R Foundation for Statistical Computing, Vienna, Austria) was used for statistical analyses and figures.

## Results

### Demographic characteristics of benchmark patients

From a patient cohort of 9356 oncological gastrectomies performed at 43 centres from five continents (Europe, Asia, North America, South America, and Africa; see *Appendix S3*), 1569 low-risk patients (16.8%) from 32 centres (*Table S1*) with a median age of 55 years undergoing TG (498 patients) and DG (1071 patients) were selected according to the benchmark criteria. Minimally invasive (laparoscopic or robotic) surgery (MIS) was performed in 66.7% (TG 41.7% and DG 78.4%; *P* < 0.001), with higher rates for early tumour stages, females, and East Asian patients (all *P* < 0.001). Overall and procedure-specific baseline characteristics of benchmark patients are detailed in *Table 1*.

**Table 1:** Baseline demographic data of the benchmark cohort, stratified by type of surgery

	Total Gastrectomy (*n* = 498)	Distal Gastrectomy (*n* = 1071)	*P*	Total (*n* = 1569)
Age (years), median (i.q.r.)	54 (47–60)	55 (48–60)	0.137	55 (48–60)
**Sex**				
Female	37.3	39.9	0.348	39.1
Male	62.7	60.1		60.9
BMI (kg/m^2^), median (i.q.r.)	23.5 (21.2–25.5)	23.3 (21.1–25.5)	0.313	23.3 (21.1–25.5)
**ASA grade**				
I	51.0	70.6	<0.001	64.4
II	49.0	29.4		35.6
**World region**				
America	10.2	4.5	<0.001	6.3
East Asia	45.0	79.1		68.3
Europe	44.8	16.4		25.4
**pTNM staging**				
0/I	37.9	65.0	<0.001	56.4
II	29.7	19.2		22.6
III	32.3	15.8		21.0
IV	0	0		0
**Tumour localization**				
Cardia/OGJ/fundus	33.3	0	<0.001	10.6
Corpus	52.0	48.3		49.5
Antrum/pylorus	10.6	51.2		38.3
Multiple, whole, linitis plastica	4.0	0.6		1.7
**Surgical access**				
Open	55.6	20.4	<0.001	31.5
Laparoscopic	37.3	66.5		57.2
Robotic	4.4	11.9		9.5
Conversion (laparoscopic to open)	2.6	1.3		1.7
**Preoperative chemotherapy**	41.0	11.6	<0.001	20.9

Values are % apart from those for age and BMI. i.q.r., interquartile range; OGJ, oesophagogastric junction.

### Perioperative outcomes for benchmark patients

The median postoperative duration of hospital stay was 10 days after both TG (i.q.r. 8–13) and DG (i.q.r. 8–11) and the median number of lymph nodes resected was 41 (i.q.r. 29–54) in TG and 39 (i.q.r. 30–50) in DG. R0 resection rates were 96.6% for TG and 98.9% for DG. Detailed perioperative outcomes for benchmark patients are shown in *Table 2*.

**Table 2:** Postoperative outcomes for the benchmark cohort, stratified by type of surgery

	Total gastrectomy (*n* = 498)	Distal gastrectomy (*n* = 1071)	*P*	Total (*n* = 1569)
Duration of hospital stay (days), median (i.q.r.)	10 (8–13)	10 (8–11)	0.023	10 (8–12)
Lymph nodes resected (*n*), median (i.q.r.)	41 (29–54)	39 (30–50)	0.001	40 (30–51)
**Resection margin**				
R0	96.6	98.9	0.005	98.2
R1	3.0	0.7		1.5
R2	0.4	0.4		0.3
Blood transfusion	3.8	2.1	0.063	2.7
Escalation of care	3.2	0.7	<0.001	1.5
Reoperation	4.2	0.7	<0.001	1.8
Readmission	3.4	1.2	0.002	1.9
**Overall morbidity**				
None	78.7	86.2	<0.001	83.8
Minor (CD grade I–II)	10.4	7.8		8.7
Major (CD grade ≥IIIA)	10.8	6.0		7.5
CCI*, median (i.q.r.)	26.2 (20.9–29.1)	20.9 (20.9–26.2)	0.001	20.9 (20.9–26.2)
**Specific complications**				
Anastomotic leakage	3 (O-J)	0.5 (G-J)		2.7
Duodenal stump leakage	1	0.2		0.4
Pancreatic fistula	0.6	0.7		0.7
Lymphatic fistula	1.2	0.7		0.8
Ileus	0.6	2		1.5
Pneumonia/pulmonary complications	3.8	0.7		1.7
Wound infections	3.4	2.2		2.5
Fluid collections	2.8	1.4		1.8
**Mortality**				
30-day	0.8	0.1	0.038	0.3
90-day	1	0.3	0.117	0.5

Values are % apart from those for duration of hospital stay, lymph nodes resected, and CCI. *Depicted CCI values are calculated only in patients with complications. i.q.r., interquartile range; CD, Clavien–Dindo; CCI, comprehensive complication index; O-J, oesophagojejunostomy; G-J, gastrojejunostomy.

In the benchmark cohort, 265 complications in 243 patients were recorded, accounting for an overall morbidity rate of 16.2% (TG 21.3% and DG 13.8%; *P* < 0.001). The rate of major complications (CD grade ≥ IIIA) was higher after TG (10.8%) than after DG (6.0%) with a median CCI of 26.2 (i.q.r. 20.9–29.1) after TG and 20.9 (i.q.r. 20.9–26.2) after DG. The 30- and 90-day mortality rates were 0.3% and 0.5% respectively. The most common complications in benchmark patients were anastomotic leakage (occurring in 2.7% (3% oesophagojejunostomy after TG and 0.5% gastrojejunostomy after DG)), wound infections (occurring in 2.5%), abdominal fluid collections (occurring in 1.8%), pneumonia/pulmonary complications (occurring in 1.7%), ileus (occurring in 1.5%), and lymphatic (0.8%) and pancreatic (0.7%) fistulae. Duodenal stump leakage occurred in 0.4% (1% after TG and 0.2% after DG). *Table 2* shows the complication rates for the surgical procedures.

### Benchmark *versus* non-benchmark patients

In the overall cohort, East Asian patients were younger, had a lower BMI, had fewer co-morbidities (ASA grade ≤ II), and had earlier-stage tumours (all *P* < 0.001) compared with European and American patients, resulting in a higher proportion of benchmark patients from East Asia (20.6%) than from Europe and America (12.0%; *Table S2*). Consequently, the benchmark cohort consisted of a higher proportion of East Asian patients (1071 patients; 68.3%), patients who were younger, a higher proportion of women, and patients who had earlier-stage tumours than the non-benchmark cohort (*Table S3*). Relevant endpoints, such as oncological parameters and rates of readmission, escalation of care, and mortality, were significantly worse for non-benchmark patients (*Table S4*). Likewise, rates of specific complications, such as anastomotic leakage (overall 4.9%, TG 6.1%, and DG 1.6%) and duodenal stump leakage (overall 1.7%, TG 1.9%, and DG 1.5%), were higher in non-benchmark patients. Wound infections were the most frequent complications in the non-benchmark cohort (6.1%) and pneumonia/pulmonary complications (5.7% overall) were significantly more frequent in Europe/America (8.3%) compared with East Asia (2.4%) and in TG (9.7%) compared with DG (2.8%).

**Fig. 1:**
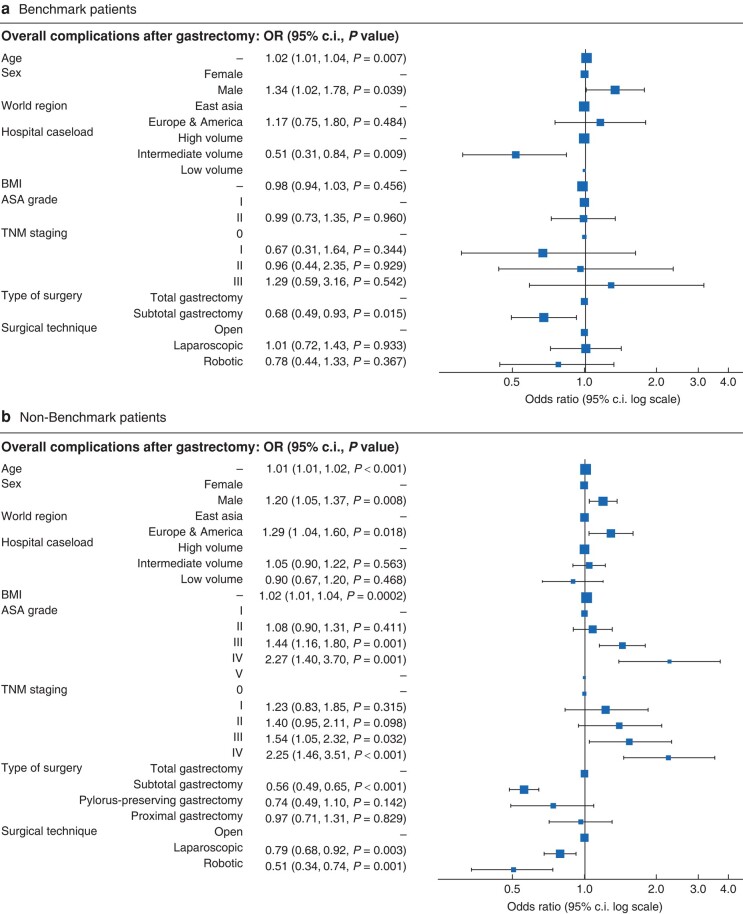
Factors prediciting occurence of complications after gastrectomy **a** OR plot of multivariable logistic regression model assessing predictive factors for occurrence of overall complications after gastrectomy in benchmark patients (1569 patients). **b** OR plot of multivariable logistic regression model assessing predictive factors for occurrence of overall complications after gastrectomy in non-benchmark patients (7787 patients).

Multivariable logistic regression revealed a significant association of overall complication rates with older age and male sex in both benchmark and non-benchmark patients. In contrast, centre localization, tumour stage, and surgical technique did not influence the morbidity of benchmark patients, whereas non-benchmark patients from East Asian centres or with early tumour stages had lower complication rates (*Fig. 1*). Whilst there was an overall association of MIS with reduced complication rates, a sensitivity analysis revealed that MIS was associated with decreased complications in East Asian centres only. In contrast, complication rates for European/American centres were associated with higher co-morbidity, but not with MIS (*Fig. S1*). Furthermore, besides fewer co-morbidities, surgery performed at an East Asian centre was the strongest predictor of favourable outcomes in terms of major complication and mortality rates (*Fig. S2*).

### Benchmark values

In addition to procedure-specific global benchmark cut-offs for TG and DG, separate regional benchmarks for East Asia and Europe/America were calculated, to account for the demographic differences between the European/American and East Asian patient cohorts outlined above.

For TG, benchmark values for duration of hospital stay were ≤ 11 days, with relevant differences between East Asia and Europe/America regarding R0 resection rate (≥ 99.4% *versus* ≥ 91.6% respectively) and lymph node yield (≥ 49 *versus* ≥ 26 respectively). Furthermore, complication-associated cut-offs, such as those for rates of blood transfusion (≤ 8.3% *versus* ≤ 1.0% respectively), escalation of care (≤ 7.1% *versus* ≤ 1.0% respectively), reoperation (≤ 8.1% *versus* 0% respectively), and readmission (≤ 7.6% *versus* ≤ 1.8% respectively), were less demanding for European/American centres compared with East Asian cut-offs. Interestingly, the benchmark values for overall (≤ 20.0% for European/American centres *versus* ≤ 21.2% for East Asian centres) and major (≤ 9.3% for European/American centres *versus* ≤ 11.3% for East Asian centres) complication rates were comparable between the two regions; however, the CCI cut-off was markedly increased in European/American centres compared with East Asian centres (≤ 51.4 *versus* ≤ 24.8 respectively). In contrast, no prominent difference in achievable cut-offs between Eastern and Western centres was observed for DG, with the exception of a higher number of lymph nodes to be resected in East Asia (≥ 39 *versus* ≥ 24). Lastly, 30- and 90-day mortality rates for both interventions should be 0% globally (see *Table 3* for procedure-specific and subdivided benchmarks for East Asia *versus* Europe/America for TG and DG).

**Table 3:** Benchmark values for oncological gastrectomy

	Total gastrectomy			Distal gastrectomy		
	Global (*n* = 498)	East Asia (*n* = 224)	Europe/America (*n* = 264)	Global (*n* = 1071)	East Asia (*n* = 847)	Europe/America (*n* = 222)
Duration of hospital stay (days)	≤11	≤11	≤11	≤10	≤10	≤10
R0 resection	≥91.6	≥99.4	≥91.6	≥99.8	≥99.2	100
Lymph node yield (*n*)	≥27	≥49	≥26	≥24	≥39	≥24
Blood transfusion	≤8.3	≤1.0	≤8.3	0	≤2.0	0
Escalation of care	≤6.6	≤1.0	≤7.1	0	≤0.6	0
Reoperation	≤7.8	0	≤8.1	0	≤0.2	0
Readmission	≤7.4	≤1.8	≤7.6	≤0.2	≤0.4	0
Any complications	≤20.0	≤21.2	≤20.0	≤15.1	≤14.8	≤14.6
Major complications	≤10.0	≤11.3	≤9.3	≤3.4	≤6.5	≤2.2
CCI*	≤33.7	≤24.8	≤51.4	≤26.2	≤24.8	≤28.0
Mortality, 30-day	0	0	0	0	0	0
Mortality, 90-day	0	0	0	0	0	0

Values are % apart from those for duration of hospital stay, lymph node yield, and CCI. *Depicted CCI values are calculated only in patients with complications. CCI, comprehensive complication index.

### Validation of benchmark values

The global benchmark values for TG were validated in defined subgroups of the total cohort: patients fulfilling all benchmark criteria, but being older than 65 years (998 patients) or having one or more 1 major co-morbidity (*Appendix S2*; 505 patients), undergoing D3 lymphadenectomy (48 patients), being treated with HIPEC (105 patients), undergoing simultaneous splenectomy (203 patients), or undergoing pancreatic/multivisceral resection (400 patients). Patient cohorts with extended benchmark criteria (older than 65 years or major co-morbidities) were within benchmark cut-offs, except for slightly elevated rates of overall and major complications, indicating that acceptable outcomes are achievable in high-volume centres, even for ‘suboptimal’ patients. In contrast, the four patient cohorts with extended surgical procedures strongly exceeded benchmark thresholds for duration of hospital stay, R0 resection rates, morbidity-associated parameters, and mortality rates (*Table 4*), confirming that the benchmark values of the present study are sensitive to extended resections performed in patients with increased co-morbidity and advanced tumour stage.

**Table 4:** Validation of benchmark values in different cohorts

	Benchmark value (TG global)	Benchmark cohort (*n* = 1796)	Non-benchmark (>65 years) (*n* = 998)	Non-benchmark (co-morbidities) (*n* = 505)	D3 LAD (*n* = 48)	HIPEC (*n* = 105)	Splenectomy (*n* = 203)	Pancreatic/multivisceral resection (*n* = 400)
Duration of hospital stay (days), median (i.q.r.)	≤11	10 (9–11)	10 (9–13)	10 (9–13)	13 (10–19)	14 (10–21)	12 (9–19)	12 (8–19)
R0 resection	≥91.6	98.4	98.5	99.2	97.9	74.3	91.1	85.8
Lymph node yield (*n*), median (i.q.r.)	≥27	40 (30–50)	36 (26–48)	34 (26–45)	31 (23–42)	38 (26–46)	46 (28–59)	33 (23–46)
Blood transfusion	≤8.3	2.4	5.6	6.1	6.3	21.0	17.2	22.3
Escalation of care	≤6.6	1.3	2.4	4.0	4.2	8.6	9.4	9.5
Reoperation	≤7.8	1.6	2.3	2.4	8.3	13.3	7.4	12.8
Readmission	≤7.4	1.7	2.3	1.8	0	10.5	3.0	7.3
Any complications	≤20.0	16.3	22.5	19.8	43.8	60.0	42.4	45.3
Major complications	≤10.0	7.5	12.2	12.5	18.8	35.2	23.2	25.0
CCI*, median (i.q.r.)	≤33.7	20.9 (20.9–26.2)	26.2 (20.9–33.5)	26.2 (20.9–26.2)	26.2 (20.9–33.7)	26.2 (20.9–41.1)	26.2 (20.9–37.1)	26.2 (20.9–42.4)
Mortality, 30-day	0	0.3	0.3	0.4	2.1	2.9	2.5	4.5
Mortality, 90-day	0	0.4	0.3	0.6	2.1	4.8	3.4	6.3

Values are % apart from those for duration of hospital stay, lymph node yield, and CCI. *Depicted CCI values are calculated only in patients with complications. TG, total gastrectomy; LAD, lymphadenectomy; HIPEC, hyperthermic intraperitoneal chemotherapy; i.q.r., interquartile range; CCI, comprehensive complication index.

## Discussion

In recent years there has been growing emphasis on the assessment of surgical outcomes to evaluate the quality of care provided by hospitals, departments, and individual surgeons. These data are often made public and used to compare the performance of different providers to improve transparency and patient autonomy^33^. This information has significant economic implications for healthcare providers, as it is often used by healthcare insurers, private payers, policymakers, the media, physicians, and patients to guide decisions. As a result, the assessment of the quality of patient care has become an important aspect of the healthcare sector’s efforts regarding public relations. However, there are concerns that the evidence provided by large national databases, audits, and meta-analyses may be biased due to the heterogeneity of the target populations and procedures analysed^34,35^. To increase sample sizes, inclusion criteria for these studies are often broad and cases are not risk adjusted, resulting in ranking systems that are inaccurate and misunderstood. In addition, this may lead to risk aversion among healthcare providers who may avoid highly morbid or complex cases^36^. Other weaknesses of traditional databases include the lack of uniform data sets, the absence of consistent validation methods, and the focus on single outcome parameters, such as the 30-day mortality rate^2^.

Benchmarking is a method for comprehensive evaluation of surgical procedures and has not previously been used in gastric cancer surgery. The aim of the present study was to address the limitations of previous research by providing benchmarks for multiple clinically relevant endpoints that can be used by third-party institutions. A particular strength of the present study is that it is based on the largest international cohort of patients undergoing oncological gastrectomy at high-volume institutions across several continents. Restriction to expert centres with comprehensive prospective gastrectomy databases and systematic classification of postoperative complications results in a high-quality data set with only minimal missing data. Using a highly selected group of patients with low co-morbidity, this approach allows the definition of global benchmark parameters, or ‘best achievable’ results, for TG and DG for the first time. Furthermore, as patient populations with gastric cancer show pronounced differences between East Asia and Europe/America, the calculation of separate benchmark values accounting for regional differences is an important aspect of the present study^37,38^. The values show considerable regional differences and it is evident that, given higher rates of co-morbidities, more advanced tumour stages, and differences in caseload and centralization, the same outcomes cannot be expected in European/American centres compared with high-volume East Asian centres. The application of the respective regional benchmark is therefore advocated as a realistic and achievable outcome metric.

The postoperative morbidities of benchmark patients (overall 16.2% and major 7.5%) and the whole study cohort (overall 25.2% and major 13.3%) are consistent with the published literature. RCTs from Korea reported overall morbidity rates between 13% and 24%^28,29,39^, which are similar to outcome data from retrospective Western and Eastern series^10,40^. On the other hand, three European RCTs^41–43^ reported considerably higher overall morbidity (34%–44%). Major morbidity in the benchmark cohort is lower than in European RCTs, but higher than in patients with locally advanced cancer from East Asian high-volume centres^44,45^. Nevertheless, the global benchmark cut-offs of the present study for overall morbidity (≤ 20%) and CCI (≤ 33.7) in TG are comparable to rates of postoperative morbidity in retrospective European series^10^, whilst benchmark values for readmission rate (≤ 7.4%), lymph node yield (≥ 27), and R0 resection rate (≥ 91.6%) are similar to the 9.1%–9.6% readmission rate, 29 lymph nodes, and 95% R0 resection rate reported in the LOGICA^41^ trial, suggesting that the global and European/American benchmarks in particular represent generally achievable goals in unselected Western patient cohorts.

Anastomotic leakage, the most frequent complication in benchmark patients, is still one of the most dreaded complications after gastrectomy, with a reported incidence between 6% and 10%^41,42,46^ and a mortality rate of up to 25%^11^ in Western centres. With respect to overall and major morbidity, it is assumed that the lower rates for the current data set (whole cohort 4.9% and benchmark cohort 2.7%) are due to an important contribution of East Asian centres to the data set, as the rate of anastomotic insufficiency in the cohort of the present study was lower in East Asian patients (2.6%) than European patients (7.0%), similar to previous reports^29,47^. Likewise, the low rate of pneumonia/pulmonary complications (1.7%) in benchmark patients is due to a low incidence in East Asian patients (benchmark: 0.8%, overall: 2.4%) compared with European/American patients (benchmark: 3.6%, overall: 8.3%). Importantly, however, these different rates are in line with published results of pneumonia in retrospective series of unselected European patients (8.3%)^10^ or prospective European RCTs, such as LOGICA (11.3%)^41^ and STOMACH (6.1%–8.5%)^42^, as well as published rates of high-quality prospective East Asian RCTs, such as JCOG0912 (0.9%)^44^, JCOG1001 (2%)^48^, KLASS-01 (0.7%–1.6%)^28^, and KLASS-02 (2.7%–3.5%)^29^. Under-reporting of these specific complications is therefore unlikely. Furthermore, rates of other specific complications, such as bowel obstruction, are within the range of published results^49^.

In Europe, the mortality rate after gastrectomy ranges between 3% and 5% in expert institutions and up to 20% in low-volume centres^8,30,41,42^. Mortality rates of the cohort in the present study are considerably lower for both benchmark and non-benchmark patients, which again are partially explained by a relevant contribution from East Asian centres where the mortality rate is typically less than 1%^28,29,50^, even in advanced cancer. Nevertheless, the low 30- and 90-day mortality rates for the whole cohort (1.2% and 1.8% respectively) also reflect the high quality of postoperative care and effective complication management in contributing Western centres and show that optimal results can be achieved in expert centres from all over the world, regardless of tumour stage and surgical approach.

Validation of the benchmark values of the present study confirms a good correlation between surgical invasiveness and morbidity, confirming previous findings from RCTs investigating the role of splenectomy^51^, extended lymphadenectomy^52^, or HIPEC^53^ in gastric cancer surgery.

There are several limitations to the present study. First, there is substantial variability in the number of cases included per centre. Due to the increased incidence of gastric cancer in East Asia and centralization of services, caseloads from the centres in Seoul and Tokyo are considerably higher than the remaining institutions (mainly in Europe and America), which might make the analysis prone to bias. Whilst this variability may be considered a strength, better reflecting reality than a single high-volume experience, it may also indicate that differences in experience with a specific procedure and learning curve-related morbidity can have an impact on the data set. In this context, it is important to emphasize that separate benchmarks for East Asian and European/American centres were calculated, to account for underlying differences in patient demographics and centre volume. Furthermore, no correlation between morbidity and centre volume was found for benchmark patients, whereas major complication and mortality rates were lower for East Asian non-benchmark patients.

Second, misclassification or under-reporting of complications could be another source of error. CD grade 1 complications are not evenly distributed among centres, suggesting that minor morbidities, such as urinary tract infections, are under-reported. However, CD grade 1 complications only have a minimal effect on the CCI and will therefore not significantly affect the results of the present study. In addition, it cannot be excluded that different definitions of complications were used by the participating centres. Certain complications, such as ileus or lymphatic fistula, are not recorded in the GASTRODATA data set and might therefore be under-reported. The reported rates of specific complications, such as pneumonia and anastomotic insufficiency, however, compare well with rates published in the literature; therefore, it is not thought that systematic under-reporting of relevant complications occurred in the retrospective data collection of the present study.

In conclusion, this study is the first to provide benchmark values for outcomes after oncological gastrectomy with lymphadenectomy. The data were collected from a large group of patients undergoing surgery in expert centres from various countries. By analysing a subgroup of ‘ideal’ patients with low co-morbidity, ‘best possible’ results for TG and DG were obtained. It is important to note that the results of the present study represent a snapshot of the current situation and that benchmark values for gastrectomy may change upon wider adoption of minimally invasive and particularly robotic techniques. Nevertheless, the results of the present study can be used as a reference due to the novelty and the relevance of the benchmark concept and the lack of comparable outcome values in the current literature.

## Collaborators

**GastroBenchmark Consortium**

Bas P. L. Wijnhoven (Department of Surgery, Erasmus University Medical Center, Rotterdam, The Netherlands); Hidde Overtoom (Department of Surgery, Erasmus University Medical Center, Rotterdam, The Netherlands); Ines Gockel (Department of Visceral, Transplant, Thoracic and Vascular Surgery, University Hospital of Leipzig, Leipzig, Germany); René Thieme (Department of Visceral, Transplant, Thoracic and Vascular Surgery, University Hospital of Leipzig, Leipzig, Germany); Ewen A. Griffiths (Department of Upper GI Surgery, Queen Elizabeth Hospital, University Hospitals Birmingham NHS Foundation Trust, Birmingham, UK); William Butterworth (Department of Upper GI Surgery, Queen Elizabeth Hospital, University Hospitals Birmingham NHS Foundation Trust, Birmingham, UK); Henrik Nienhüser (Klinik für Allgemein-, Viszeral- und Transplantationschirurgie, Universitätsklinikum Heidelberg, Heidelberg, Germany); Beat Müller (Klinik für Allgemein-, Viszeral- und Transplantationschirurgie, Universitätsklinikum Heidelberg, Heidelberg, Germany); Nerma Crnovrsanin (Klinik für Allgemein-, Viszeral- und Transplantationschirurgie, Universitätsklinikum Heidelberg, Heidelberg, Germany); Felix Nickel (Department of General, Visceral, and Thoracic Surgery, University Medical Center Hamburg-Eppendorf, Hamburg, Germany); Suzanne Gisbertz (Amsterdam University Medical Center & Cancer Center Amsterdam, University of Amsterdam, Department of Surgery, Amsterdam, The Netherlands); Mark van Berghe Henegouwen (Amsterdam University Medical Center & Cancer Center Amsterdam, University of Amsterdam, Department of Surgery, Amsterdam, The Netherlands); Philip H. Pucher (Department of Surgery, Queen Alexandra Hospital, Portsmouth Hospitals NHS Trust, Portsmouth, UK); Kashuf Khan (Department of Surgery, Queen Alexandra Hospital, Portsmouth Hospitals NHS Trust, Portsmouth, UK); Asif Chaudry (The Royal Marsden NHS Foundation Trust, Chelsea, London, SW3 6JJ, UK); Pranav H. Patel (The Royal Marsden NHS Foundation Trust, Chelsea, London, SW3 6JJ, UK); Manuel Pera (Section of Gastrointestinal Surgery, Hospital Universitario del Mar, Universitat Autònoma de Barcelona, Barcelona, Spain); Mariagiulia Dal Cero (Section of Gastrointestinal Surgery, Hospital Universitario del Mar, Universitat Autònoma de Barcelona, Barcelona, Spain); Carlos Garcia (Hospital San Borja Arriarán, Av. Sta. Rosa 1234, Santiago, Región Metropolitana, Chile); Guillermo Martinez Salinas (Hospital San Borja Arriarán, Av. Sta. Rosa 1234, Santiago, Región Metropolitana, Chile); Paulo Kassab (Gastroesophageal and Bariatric Surgical Division, Department of Surgery, Santa Casa of São Paulo Medical School and Hospital, São Paulo, Brazil); Osvaldo Antônio Prado Castro (Gastroesophageal and Bariatric Surgical Division, Department of Surgery, Santa Casa of São Paulo Medical School and Hospital, São Paulo, Brazil); Enrique Norero (Esophagogastric Surgery Unit, Digestive Surgery Department, Hospital Dr Sotero del Rio, Pontificia Universidad Catolica de Chile, Santiago, Chile); Paul Wisniowski (Division of Upper GI and General Surgery, Keck School of Medicine, University of Southern California, 1510 San Pablo St., Health Sciences Campus, Los Angeles, USA); Luke Randall Putnam (Division of Upper GI and General Surgery, Keck School of Medicine, University of Southern California, 1510 San Pablo St., Health Sciences Campus, Los Angeles, USA); Pietro Maria Lombardi (Division of Minimally Invasive Surgical Oncology, Niguarda Cancer Center, ASST Grande Ospedale Metropolitano Niguarda, Piazza Ospedale Maggiore, 3, 20162, Milan, Italy); Giovanni Ferrari (Division of Minimally Invasive Surgical Oncology, Niguarda Cancer Center, ASST Grande Ospedale Metropolitano Niguarda, Piazza Ospedale Maggiore, 3, 20162, Milan, Italy); Rita Gudaityte (Department of Surgery, Hospital of Lithuanian University of Health Sciences, Eiveniu 2, Kaunas 50161, Lithuania); Almantas Maleckas (Department of Surgery, Hospital of Lithuanian University of Health Sciences, Eiveniu 2, Kaunas 50161, Lithuania); Leanne Prodehl (Department of Surgery, Charlotte Maxeke Johannesburg Academic Hospital, University of the Witwatersrand, Johannesburg, South Africa); Antonio Castaldi (Service de Chirurgie Digestive et Cancérologie Digestive, Hôpital Universitaire Carémeau, Nîmes, France); Michel Prudhomme (Service de Chirurgie Digestive et Cancérologie Digestive, Hôpital Universitaire Carémeau, Nîmes, France); Simone Giacopuzzi (Department of Surgery, University Hospital of Verona, Verona, Italy); Francesco Puccetti (Department of Surgery, San Raffaele Hospital, Milano, Italy); Domenico D'Ugo (FONDAZIONE POLICLINICO UNIVERSITARIO GEMELLI-IRCCS, Roma, Italy); Daniel Gero (Department of Surgery & Transplantation, University Hospital Zürich, Raemistrasse 100, 8091 Zurich, Switzerland); Hyuk-Joon Lee (Department of Surgery, Seoul National University Cancer Hospital, 101 Daehak-ro Jongno-gu, Seoul, South Korea).

**GASTRODATA Consortium**

Guillaume Piessen (Department of Surgery, University Hospital of Lille, Lille, France); Justine Lerooy (Department of Surgery, University Hospital of Lille, Lille, France); Johanna Wilhelmina van Sandick (Department of Surgical Oncology, The Netherlands Cancer Institute—Antoni van Leeuwenhoek Hospital, Postbus, 90203 1006 BE, Amsterdam, The Netherlands); John V. Reynolds (Department of Surgery, St. James’s Hospital, Trinity College Dublin, Dublin, Ireland); Paolo Morgagni (GB Morgagni-L Pierantoni Hospital, Forlì, Italy); Arnulf H. Hölscher (Contilia Center for Esophageal Diseases, Elisabeth Hospital Essen, West German Tumor Center, University Medicine Essen, Germany); Martin Hemmerich (Contilia Center for Esophageal Diseases, Elisabeth Hospital Essen, West German Tumor Center, University Medicine Essen, Germany); Stefan Mönig (Department of Surgery, University Hospital of Geneva, Geneva, Switzerland); Mickael Chevallay (Department of Surgery, University Hospital of Geneva, Geneva, Switzerland); Piotr Kołodziejczyk (Department of Surgery, Jagiellonian University, Kraków, Poland); Henk Hartgrink (Leiden University Medical Center, Leiden, The Netherlands); Paulo Matos da Costa (Faculdade de Medicina, Universidade de Lisboa; Lisboa, Portugal); Filipe Castro Borges (Faculdade de Medicina, Universidade de Lisboa; Lisboa, Portugal); Andrew Davies (Department of Surgery, Guy’s & St Thomas’ NHS Foundation Trust, London, UK); Cara Baker (Department of Surgery, Guy’s & St Thomas’ NHS Foundation Trust, London, UK); William Allum (The Royal Marsden NHS Foundation Trust, Chelsea, London, SW3 6JJ, UK); Sacheen Kumar (The Royal Marsden NHS Foundation Trust, Chelsea, London, SW3 6JJ, UK); Wojciech Polkowski (Medical University of Lublin, Lublin, Poland); Karol Rawicz-Pruszyński (Medical University of Lublin, Lublin, Poland); Uberto Fumagalli Romario (Digestive Surgery, European Institute of Oncology, IRCCS, Milano, Italy); Stefano De Pascale (Digestive Surgery, European Institute of Oncology, IRCCS, Milano, Italy); Antonio Tarasconi (Department of Surgery, University Hospital of Brescia, Brescia, Italy); Daniel Reim (Department of Surgery, TUM School of Medicine, Technical University of Munich, Germany); Ilaria Pergolini (Department of Surgery, TUM School of Medicine, Technical University of Munich, Germany); Lucio Lara Santos (Department of Surgery, Portuguese Institute of Oncology, Porto, Portugal); Pedro Carvalho Martins (Department of Surgery, Portuguese Institute of Oncology, Porto, Portugal); Alberto Biondi (FONDAZIONE POLICLINICO UNIVERSITARIO GEMELLI-IRCCS, Roma, Italy); Maurizio Degiuli (Department of Surgical Oncology and Digestive Surgery, San Luigi University Hospital Orbassano, School of Medicine, University of Torino, Torino, Italy); Rossella Reddavid (Department of Surgical Oncology and Digestive Surgery, San Luigi University Hospital Orbassano, School of Medicine, University of Torino, Torino, Italy); Wojciech Kielan (Wroclaw Medical University, Wroclaw, Poland); Paul Magnus Schneider (Digestive Oncology Tumor Center and Esophageal Cancer Center, Hirslanden Medical Center, Zurich, Switzerland); Thomas Murphy (Mercy University Hospital, Cork, Ireland).

## Supplementary Material

znad379_Supplementary_Data

## Data Availability

All research data supporting this publication are available from the corresponding author upon reasonable request.
