# Supplementary material for: Defining benchmarks for total and distal gastrectomy: global multicentre analysis
Source: Br J Surg. 2024 Feb 20;111(2):znad379. doi: 10.1093/bjs/znad379 (PMC10878554; doi:10.1093/bjs/znad379)
Supplement: znad379_Supplementary_Data [file znad379_supplementary_data.docx]

**Defining benchmarks for total and distal gastrectomy – a global multi-centre analysis**

Marcel André Schneider^1,^*, Jeesun Kim^2,^*, Felix Berlth^3,^*, Yutaka Sugita^4^, Daniel Gero^1^, Peter P. Grimminger^3^, Bas P. L. Wijnhoven^5^, Hidde Overtoom^5^, Ines Gockel^6^, René Thieme^6^, Ewen A. Griffiths^7^, William Butterworth^7^, Henrik Nienhüser^8^, Beat Müller^8^, Nerma Crnovrsanin^8^, Felix Nickel^9^, Suzanne Gisbertz^10^, Mark van Berghe Henegouwen^10^, Philip H. Pucher^11^, Kashuf Khan^11^, Asif Chaudry^12^, Pranav H. Patel^12^, Manuel Pera^13^, Mariagiulia Dal Cero^13^, Carlos Garcia^14^, Guillermo Martinez Salinas^14^, Paulo Kassab^15^, Osvaldo Antônio Prado Castro^15^, Enrique Norero^16^, Paul Wisniowski^17^, Luke Randall Putnam^17^, Pietro Maria Lombardi^18^, Giovanni Ferrari^18^, Rita Gudaityte^19^, Almantas Maleckas^19^, Leanne Prodehl^20^, Antonio Castaldi^21^, Michel Prudhomme^21^, Hyuk-Joon Lee^2^, Takeshi Sano^4^, Gian Luca Baiocchi^22^, Giovanni De Manzoni^23^, Simone Giacopuzzi^23^, Maria Bencivenga^23^, Riccardo Rosati^24^, Francesco Puccetti^24^, Domenico D'Ugo^25^, the GASTRODATA consortium^26^, Souya Nunobe^4^, Han-Kwang Yang^2,+^ & Christian Alexander Gutschow^1,+^

**Shared first authorship, ^+^Shared last authorship*

*^1^ Department of Surgery & Transplantation, University Hospital Zürich, Raemistrasse 100, 8091 Zurich, Switzerland*

*^2^ Department of Surgery, Seoul National University Cancer Hospital, 101 Daehak-ro Jongno-gu, Seoul, South Korea*

*^3^ Department of General-, Visceral- and Transplant Surgery, University Medical Center Mainz, Langenbeckstraße 1, 55131 Mainz, Germany*

*^4^ Department of Gastroenterological Surgery, Cancer Institute Hospital of the Japanese Foundation for Cancer Research, 3-8-31, Ariake, Koto-ku, Tokyo, 135-8550, Japan*

*^5^ Department of Surgery, Erasmus University Medical Center, Rotterdam, The Netherlands*

*^6^ Department of Visceral, Transplant, Thoracic and Vascular Surgery, University Hospital of Leipzig, Leipzig, Germany*

*^7^ Department of Upper GI Surgery, Queen Elizabeth Hospital, University Hospitals Birmingham NHS Foundation Trust, Birmingham, United Kingdom*

*^8^ Klinik für Allgemein-, Viszeral- und Transplantationschirurgie, Universitätsklinikum Heidelberg, Im Neuenheimer Feld 420, 69120 Heidelberg, Germany*

*^9^ Department of General, Visceral, and Thoracic Surgery, University Medical Center Hamburg-Eppendorf, Martinistrasse 52, 20251 Hamburg, Germany*

*^10^ Amsterdam University Medical Center & Cancer Center Amsterdam, University of Amsterdam, Department of Surgery, Amsterdam, the Netherlands*

*^11^ Department of Surgery, Queen Alexandra Hospital, Portsmouth Hospitals NHS Trust, Portsmouth, United Kingdom*

*^12^ The Royal Marsden NHS Foundation Trust, Chelsea, London, SW3 6JJ, United Kingdom*

*^13^ Section of Gastrointestinal Surgery, Hospital Universitario del Mar, Universitat Autònoma de Barcelona, Barcelona, Spain*

*^14^ Hospital San Borja Arriarán, Av. Sta. Rosa 1234, Santiago, Región Metropolitana, Chile*

*^15^ Gastroesophageal and Bariatric Surgical Division, Department of Surgery, Santa Casa of São Paulo Medical School and Hospital, São Paulo, Brazil*

*^16^ Esophagogastric Surgery Unit, Digestive Surgery Department, Hospital Dr Sotero del Rio, Pontificia Universidad Catolica de Chile, Santiago, Chile*

*^17^ Division of Upper GI and General Surgery, Keck School of Medicine, University of Southern California, 1510 San Pablo St., Health Sciences Campus, Los Angeles, USA*

*^18^ Division of Minimally Invasive Surgical Oncology, Niguarda Cancer Center, ASST Grande Ospedale Metropolitano Niguarda, Piazza Ospedale Maggiore, 3, 20162, Milan, Italy*

*^19^ Department of Surgery, Hospital of Lithuanian University of Health Sciences, Eiveniu 2, Kaunas 50161, Lithuania*

*^10^Department of Surgery, Charlotte Maxeke Johannesburg Academic Hospital, University of the Witwatersrand, Johannesburg, South Africa*

*^21^ Service de Chirurgie Digestive et Cancérologie Digestive, Hôpital Universitaire Carémeau, Nîmes, France*

*^22^ Department of Surgery, University Hospital of Brescia, Brescia, Italy*

*^23^ Department of Surgery, University Hospital of Verona, Verona, Italy*

*^24^ San Raffaele Hospital, Milano, Italy*

*^25^ FONDAZIONE POLICLINICO UNIVERSITARIO GEMELLI-IRCCS, Roma, Italy*

*^26^ See acknowledgements for further GASTRODATA contributors and respective affiliations.*

**Corresponding author:**

Prof. Dr. med. Christian A. Gutschow

Section Head Upper Gastrointestinal Surgery

Department for General and Transplantation Surgery

University Hospital Zurich

Rämistrasse 100, CH-8091 Zurich

Phone: (+41) 044 255 9723

Mail: christian.gutschow@usz.ch

ORCID ID 0000-0001-6171-4427

Twitter @ChristianGutsc2

**Supplementary Materials - Index**

| **Supplementary Figures and Tables** |  |
| --- | --- |
| Supplementary figure 1 | *page 3* |
| Supplementary figure 2 | *page 4* |
| Supplementary table 1: Center caseload and number of benchmark patients | *page 5* |
| Supplementary table 2: Baseline data of the whole cohort, stratified by East Asian vs. European/American patients | *page 6* |
| Supplementary table 3: Baseline data of the whole cohort, stratified by benchmark vs. non-benchmark patients | *page 8* |
| Supplementary table 4: Postoperative outcomes of the whole cohort, stratified by benchmark vs. non-benchmark patients | *page 10* |
| **Supplementary Appendixes** |  |
| Appendix 1: Detailed overall patient in-/exclusion criteria | *page 12* |
| Appendix 2: Detailed benchmark in-/exclusion criteria | *page 13* |
| Appendix 3: Participating centers | *page 15* |

**Supplementary Figures and Tables**

**Supplementary figure 1**


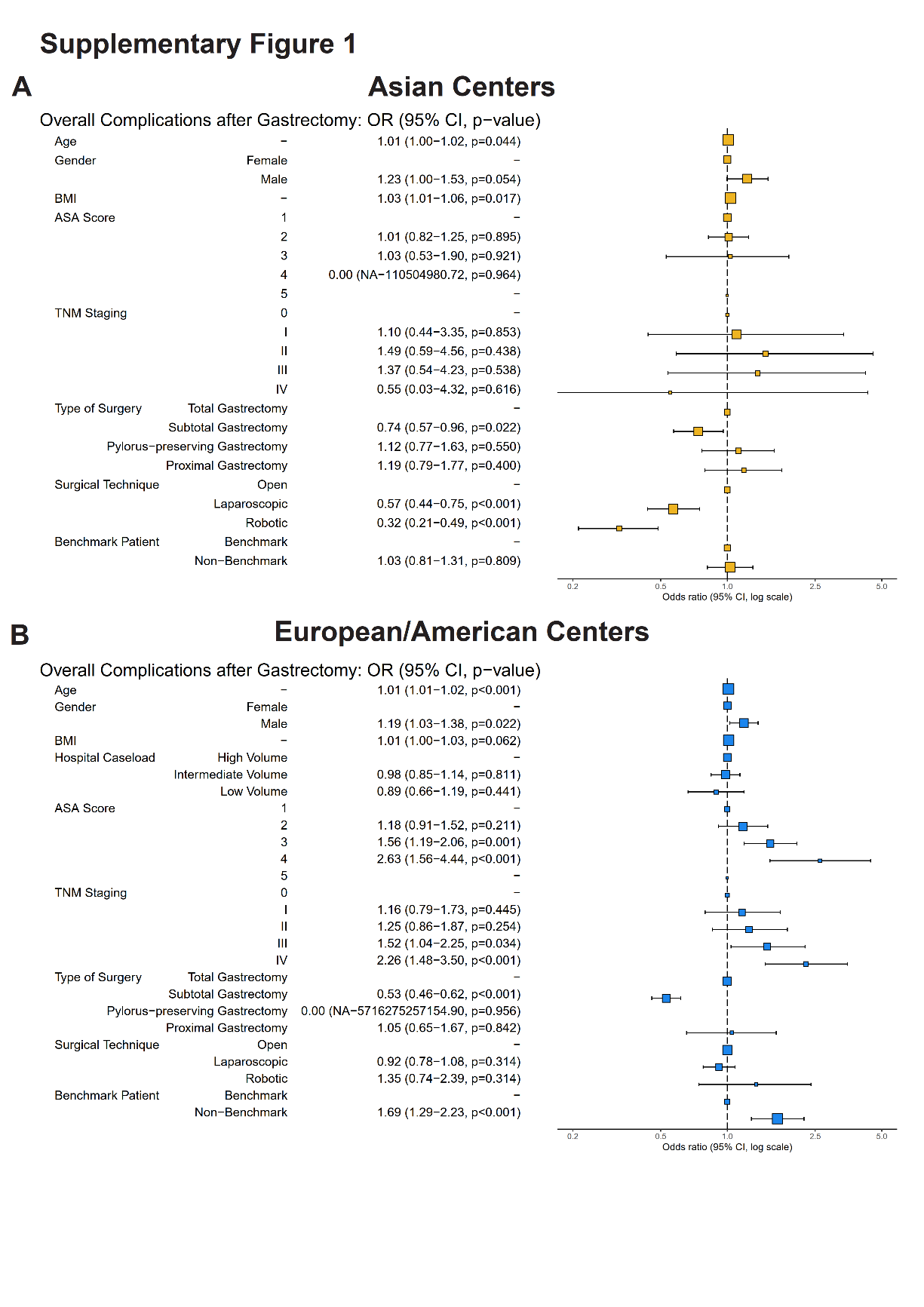


(**A**) OR plot of multivariable logistic regression model assessing predictive factors on occurrence of overall complications after gastrectomy in East Asian centers (n=5209) as part of the sensitivity analysis. (**B**) OR plot of multivariable logistic regression model assessing predictive factors on occurrence of overall complications after gastrectomy in European & American centers (n=4147) as part of the sensitivity analysis.

**Supplementary figure 2**


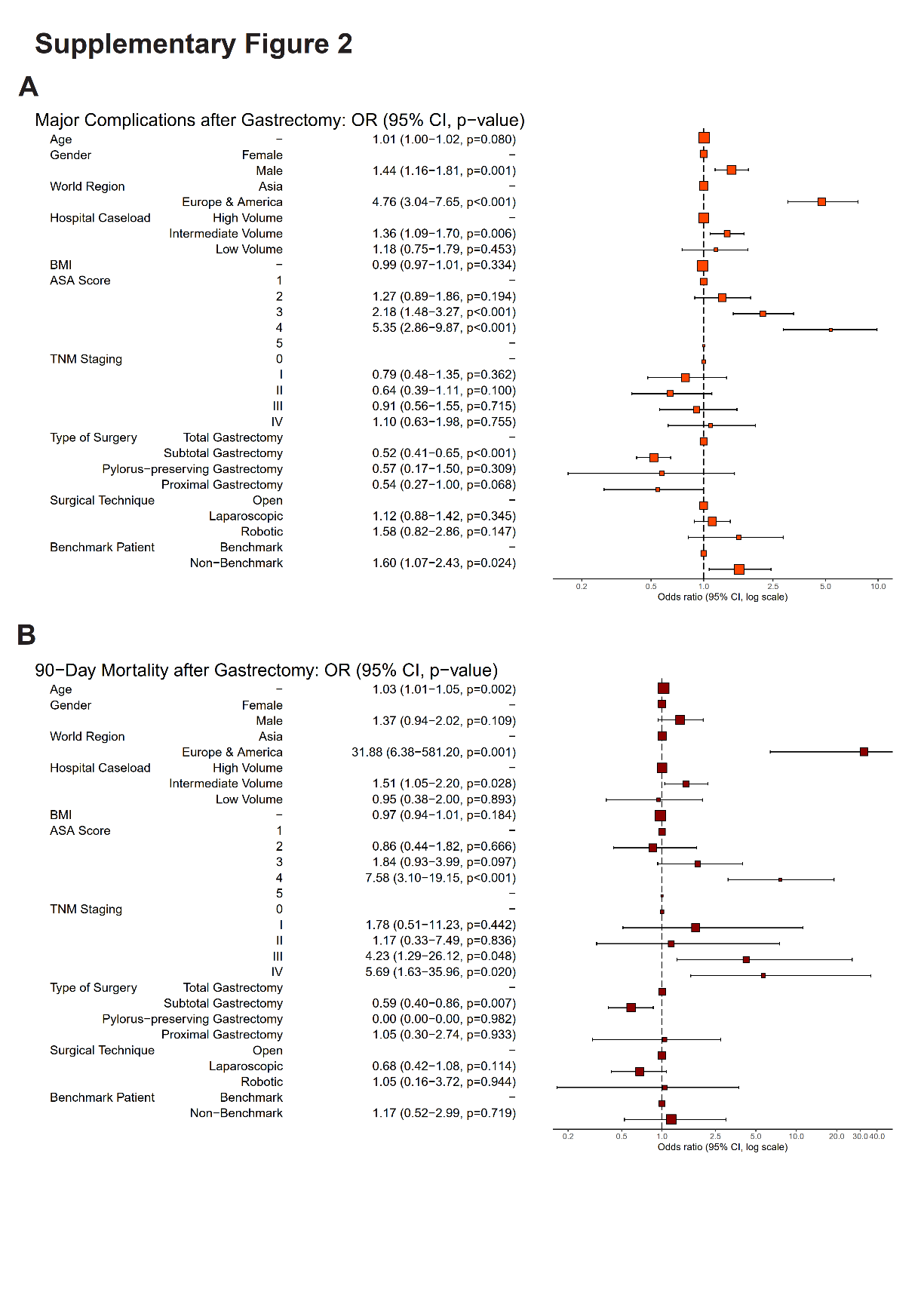


(**A**) OR plot of multivariable logistic regression model assessing predictive factors on occurrence of major complications after gastrectomy in the overall cohort (n=9356). (**B**) OR plot of multivariable logistic regression model assessing predictive factors on occurrence of 90-day mortality after gastrectomy in the overall cohort (n=9356).

**Supplementary table 1: Center caseload and number of benchmark patients**

| **Center** | **Benchmark**  **patients**  **n (%)** | **Non-Benchmark**  **patients**  **n (%)** | **Total Nr. of patients** |
| --- | --- | --- | --- |
| National University Hospital, Seoul, Republic of Korea | 679 (20.1%) | 2696 (79.9%) | 3375 |
| Cancer Institute Hospital of the JFCR, Tokyo, Japan | 392 (21.4%) | 1442 (78.6%) | 1834 |
| University Hospital, Verona, Italy | 56 (14.3%) | 335 (85.7%) | 391 |
| University Hospital, Heidelberg, Germany | 31 (11.1%) | 249 (88.9%) | 280 |
| Istituto Europeo di Oncologia, Milano, Italy | 37 (14.4%) | 220 (85.6%) | 257 |
| San Raffaele Hospital, Milano, Italy | 29 (12.6%) | 201 (87.4%) | 230 |
| Hospital Dr Sotero del Rio, Santiago, Chile | 31 (16.5%) | 157 (83.5%) | 188 |
| Academic Medical Center, Amsterdam, The Netherlands | 25 (14.5%) | 147 (85.5%) | 172 |
| GB Morgagni-L Pierantoni Hospital, Forlì, Italy | 11 (6.8%) | 151 (93.2%) | 162 |
| Queen Elizabeth Hospital, University Hospitals Birmingham, Birmingham, United Kingdom | 16 (10.7%) | 133 (89.3%) | 149 |
| Hospital of Lithuanian University of Health Sciences, Kaunas, Lithuania | 34 (23.9%) | 108 (76.1%) | 142 |
| Niguarda Hospital, Milano, Italy | 14 (10.4%) | 121 (89.6%) | 135 |
| Erasmus University Medical Center, Rotterdam, The Netherlands | 17 (12.7%) | 117 (87.3%) | 134 |
| Medical University, Lublin, Poland | 11 (8.5%) | 118 (91.5%) | 129 |
| Santa Casa Medical School, São Paulo, Brazil | 45 (34.9%) | 84 (65.1%) | 129 |
| Johannes Gutenberg University Hospital, Mainz, Germany | 11 (9.5%) | 104 (90.5%) | 115 |
| Portuguese Institute of Oncology, Porto, Portugal | 5 (4.5%) | 106 (95.5%) | 111 |
| Jagiellonian University, Kraków, Poland | 8 (8.2%) | 90 (91.8%) | 98 |
| Medical University, Wroclaw, Poland | 24 (24.7%) | 73 (75.3%) | 97 |
| University Hospital, Leipzig, Germany | 12 (14.0%) | 74 (86.0%) | 86 |
| University Hospital, Torino, Italy | 3 (3.5%) | 83 (96.5%) | 86 |
| University Hospital, Zurich, Switzerland | 10 (11.9%) | 74 (88.1%) | 84 |
| Keck Medical School, University of Southern California, Los Angeles, USA | 1 (1.2%) | 82 (98.8%) | 83 |
| Netherlands Cancer Institute, Antoni van Leeuwenhoek Hospital, Amsterdam, the Netherlands | 14 (17.3%) | 67 (82.7%) | 81 |
| Royal Marsden NHS, London, United Kingdom (Prof. A. Chaudry) | 12 (16.0%) | 63 (84.0%) | 75 |
| University Hospital, Brescia, Italy | 2 (2.7%) | 73 (97.3%) | 75 |
| St. Thomas' NHS, London, United Kingdom | 15 (22.7%) | 51 (77.3%) | 66 |
| Cattolica University, Roma, Italy | 7 (10.9%) | 57 (89.1%) | 64 |
| Hospital San Borja Arriarán, Santiago, Chile | 11 (17.7%) | 51 (82.3%) | 62 |
| Technical University, Munich, Germany | 4 (6.7%) | 56 (93.3%) | 60 |
| Queen Alexandra Hospital, Portsmouth, United Kingdom | 1 (1.9%) | 51 (98.1%) | 52 |
| University Hospital, Lisbon, Portugal | 1 (2.0%) | 50 (98.0%) | 51 |
| Hospital del Mar, Barcelona, Spain | 0 (0%) | 49 (100%) | 49 |
| Centre Hospitalier Régional, Lille, France | 0 (0%) | 40 (100%) | 40 |
| Royal Marsden NHS, London, United Kingdom (Prof. W. Allum) | 0 (0%) | 39 (100%) | 39 |
| St. James’s Hospital, Trinity College, Dublin, Ireland | 0 (0%) | 37 (100%) | 37 |
| Hôpital Universitaire Carémeau, Nîmes, France | 0 (0%) | 31 (100%) | 31 |
| Mercy University Hospital, Cork, Ireland | 0 (0%) | 31 (100%) | 31 |
| University Medical Center, Leiden, the Netherlands | 0 (0%) | 30 (100%) | 30 |
| University Hospital, Geneva, Switzerland | 0 (0%) | 20 (100%) | 20 |
| Hirslanden Medical Center, Zurich, Switzerland | 0 (0%) | 12 (100%) | 12 |
| Charlotte Maxeke Johannesburg Academic Hospital, Johannesburg, South Africa | 0 (0%) | 8 (100%) | 8 |
| Agaplesion Markus Hospital, Frankfurt, Germany | 0 (0%) | 6 (100%) | 6 |

**Supplementary table 2: Baseline data of the whole cohort, stratified by East Asian vs. European/American patients**

|  | **East Asia** **(n=5209)** | **Europe & America (n=4147)** | **P-value** | **Total (n=9356)** |
| --- | --- | --- | --- | --- |
| **Age** (Years, Median [IQR]) | 64  [55, 72] | 68  [58, 76] | <0.001 | 65  [56, 74] |
| **Gender** |  |  |  |  |
| Female | 36.5% | 38.9% | 0.013 | 37.6% |
| Male | 63.5% | 61.1% |  | 62.4% |
| **BMI** (Median [IQR]) | 23.2  [21.1, 25.5] | 25.0  [22.5, 28.1] | <0.001 | 24.0  [21.6, 26.8] |
| Missing | 34.2% | 6.0% |  | 21.7% |
| **ASA Score** |  |  |  |  |
| 1 | 43.2% | 12.2% | <0.001 | 29.5% |
| 2 | 39.9% | 49.3% |  | 44.0% |
| 3 | 4.3% | 34.2% |  | 17.6% |
| 4 | 0.3% | 2.0% |  | 1.0% |
| Missing | 12.3% | 2.3% |  | 7.9% |
| **pTNM Staging** |  |  |  |  |
| 0 | 1.1% | 4.5% | <0.001 | 2.6% |
| I | 62.7% | 27.3% |  | 47.0% |
| II | 14.9% | 25.9% |  | 19.8% |
| III | 11.6% | 31.9% |  | 20.6% |
| IV | 1.2% | 9.4% |  | 4.8% |
| Missing | 8.5% | 1.1% |  | 5.2% |
| **Lauren Classification** |  |  |  |  |
| Diffuse Type | 42.5% | 26.7% | <0.001 | 35.5% |
| Intestinal Type | 41.2% | 31.2% |  | 36.7% |
| Other | 16.3% | 42.1% |  | 27.7% |
| **Tumor Localization** |  |  |  |  |
| Antrum/Pylorus | 28.0% | 39.5% | <0.001 | 33.1% |
| Cardia/EGJ | 5.6% | 13.8% |  | 9.2% |
| Corpus | 52.6% | 43.1% |  | 48.4% |
| Fundus | 6.8% | 1.9% |  | 4.6% |
| Multiple, Whole, Linitis Plastica | 7.0% | 1.7% |  | 4.7% |
| **Type of Surgery** |  |  |  |  |
| Total Gastrectomy | 17.5% | 52.4% | <0.001 | 33.0% |
| Subtotal Gastrectomy | 58.9% | 45.6% |  | 53.0% |
| Pylorus-preserving Gastrectomy | 15.7% | 0.0% |  | 8.7% |
| Proximal Gastrectomy | 7.9% | 2.0% |  | 5.3% |
| **Surgical Access** |  |  |  |  |
| Open | 17.7% | 66.6% | <0.001 | 39.3% |
| Laparoscopic | 70.0% | 27.9% |  | 51.3% |
| Robotic | 11.4% | 1.6% |  | 7.0% |
| Conversion (Lap ->Open) | 0.8% | 3.9% |  | 2.1% |
| Conversion (Robot ->Open) | 0% | 0.1% |  | 0.1% |
| Missing | 0.2% | 0% |  | 0.1% |
| **Preoperative Chemotherapy** | 4.3% | 46.7% | <0.001 | 23.1% |
| **Benchmark Patient** | 20.6% | 12.0% | <0.001 | 16.8% |
| Table legend: IQR: Interquartile range | | | | |

Supplementary table 3: Baseline data of the whole cohort, stratified by benchmark vs. non-benchmark patients

|  | **Benchmark (n=1569)** | **Non-Benchmark** **(n=7787)** | **P-value** | **Total (n=9356)** |
| --- | --- | --- | --- | --- |
| **Age** (Years, Median [IQR]) | 55  [47, 60] | 68  [59, 75] | <0.001 | 65  [56, 74] |
| **Gender** |  |  |  |  |
| Female | 39.1% | 37.3% | <0.001 | 37.6% |
| Male | 60.9% | 62.7% |  | 62.4% |
| **BMI** (Median [IQR]) | 23.3  [21.1, 25.5] | 24.3  [21.9, 27.1] | <0.001 | 24.0  [21.6, 26.8] |
| Missing | 0% | 26.0% |  | 21.7% |
| **ASA Score** |  |  |  |  |
| 1 | 64.4% | 22.4% | <0.001 | 29.5% |
| 2 | 35.6% | 45.7% |  | 44.0% |
| 3 | 0% | 21.1% |  | 17.6% |
| 4 | 0% | 1.2% |  | 1.0% |
| Missing | 0% | 9.5% |  | 7.9% |
| **World Region** |  |  |  |  |
| Americas/Africa | 6.3% | 4.8% | <0.001 | 5.0% |
| Asia | 68.3% | 53.1% |  | 55.7% |
| Europe | 25.4% | 42.1% |  | 39.3% |
| **pTNM Staging** |  |  |  |  |
| 0 | 2.7% | 2.6% | <0.001 | 2.6% |
| I | 53.7% | 45.7% |  | 47.0% |
| II | 22.6% | 19.2% |  | 19.8% |
| III | 21.0% | 20.5% |  | 20.6% |
| IV | 0% | 5.8% |  | 4.8% |
| Missing | 0% | 6.2% |  | 5.2% |
| **Lauren Classification** |  |  |  |  |
| Diffuse Type | 51.3% | 32.3% | <0.001 | 35.5% |
| Intestinal Type | 30.5% | 38.0% |  | 36.7% |
| Other | 18.2% | 29.7% |  | 27.7% |
| **Tumor Localization** |  |  |  |  |
| Antrum/Pylorus | 38.3% | 32.1% | <0.001 | 33.1% |
| Cardia/EGJ | 7.4% | 9.6% |  | 9.2% |
| Corpus | 49.5% | 48.1% |  | 48.4% |
| Fundus | 3.2% | 4.9% |  | 4.6% |
| Multiple, Whole, Linitis Plastica | 1.7% | 5.3% |  | 4.7% |
| **Type of Surgery** |  |  |  |  |
| Total Gastrectomy | 31.7% | 33.2% | <0.001 | 33.0% |
| Subtotal Gastrectomy | 68.3% | 49.9% |  | 53.0% |
| Pylorus-preserving Gastrectomy | 0% | 10.5% |  | 8.7% |
| Proximal Gastrectomy | 0% | 6.4% |  | 5.3% |
| **Surgical Access** |  |  |  |  |
| Open | 31.5% | 40.9% | <0.001 | 39.3% |
| Laparoscopic | 57.2% | 50.1% |  | 51.3% |
| Robotic | 9.5% | 6.5% |  | 7.0% |
| Conversion (Lap ->Open) | 1.7% | 2.2% |  | 2.1% |
| Conversion (Robot ->Open) | 0% | 0.1% |  | 0.1% |
| Missing | 0% | 0.1% |  | 0.1% |
| **Preoperative Chemotherapy** | 20.9% | 23.5% |  | 23.1% |
| Table legend: IQR: Interquartile range | | | | |

**Supplementary table 4: Postoperative outcomes of the whole cohort, stratified by benchmark vs. non-benchmark patients**

|  | **Benchmark (n=1569)** | **Non-Benchmark (n=7787)** | **P-value** | **Total (n=9356)** |
| --- | --- | --- | --- | --- |
| **Hospital Stay (Days,**  Median [IQR]**)** | 10  [8-12] | 10  [9, 13] | <0.001 | 10  [9, 13] |
| **Lymph Nodes resected (n,**  Median [IQR]) | 40  [30, 51] | 34  [25, 46] | <0.001 | 35  [26, 47] |
| **Resection Margin** |  |  |  |  |
| R0 | 98.2% | 95.8% | <0.001 | 96.2% |
| R1 | 1.4% | 3.3% |  | 3.0% |
| R2 | 0.4% | 0.9% |  | 0.8% |
| **Blood Transfusion** | 2.7% | 8.3% | <0.001 | 7.4% |
| **Escalation of Care** | 1.5% | 4.4% | <0.001 | 3.9% |
| **Reoperation** | 1.8% | 4.9% | <0.001 | 4.4% |
| **Surgery Related Readmissions** | 1.9% | 4.0% | <0.001 | 3.6% |
| **Overall Morbidity** |  |  |  |  |
| None | 83.8% | 73.0% | <0.001 | 74.8% |
| Minor (CD I-II) | 8.7% | 12.6% |  | 11.9% |
| Major (CD ≥IIIA) | 7.5% | 14.4% |  | 13.3% |
| **CCI***(Median [IQR]) | 20.9  [20.9-26.2] | 26.2  [20.9-33.7] | 0.001 | 26.2  [20.9-33.7] |
| **Specific Complications** |  |  |  |  |
| Anastomotic Leakage | 2.7% | 4.9% | <0.001 | 4.7% |
| Duodenal Stump Leakage | 0.4% | 1.7% | <0.001 | 1.2% |
| Pancreatic Fistula | 0.7% | 1.5% | <0.001 | 1.4% |
| Lymphatic Fistula | 0.8% | 0.6% | 0.367 | 0.6% |
| Ileus | 1.9% | 2.6% | <0.001 | 2.5% |
| Pneumonia/Pulmonary Complications | 1.7% | 5.7% | <0.001 | 5.2% |
| Wound Infections | 2.5% | 6.1% | <0.001 | 5.9% |
| Fluid Collections | 1.8% | 3.3% | <0.001 | 3.1% |
| **Mortality** |  |  |  |  |
| 30 Day | 0.3% | 1.4% | 0.002 | 1.2% |
| 90 Day | 0.5% | 2.0% | <0.001 | 1.8% |
| Table legend: TG: Total gastrectomy, DG: Distal subtotal gastrectomy, PPG: Pylorus-preserving gastrectomy, PG: Proximal gastrectomy. * Depicted CCI values are calculated only in patients with complications; E-J: Esophagojejunostomy; G-J: Gastrojejunostomy; G-G: Gastrogastrostomy; CD: Clavien-Dindo classification of complications. IQR: Interquartile range | | | | |

**Supplementary Appendixes**

**Appendix 1: Detailed overall patient in-/exclusion criteria**

Inclusion criteria:

All consecutive patients fulfilling the inclusion criteria during the mentioned period were included in the database:

- Patients with histologically proven, primary, resectable (cT1-4a, N0-3b, M0) gastric adenocarcinoma
- Undergoing elective total (defined as: complete removal of stomach), distal subtotal, proximal or pylorus-preserving, open or minimally invasive gastrectomy (laparoscopically, robotically assisted) with lymphadenectomy.
  - Minimally invasive defined as: a mini-laparotomy or a Pfannenstiel incision <8cm in both the skin and fascia performed for specimen removal
  - All reconstruction techniques can be included (Roux-en-Y +/- Pouch, Billroth II/Omega (+/- Braun), Billroth I/Delta, Jejunal-interposition (including Longmire, Henley, Merendino, double-tract reconstruction), etc.).
- Surgery between 01.01.2017 – 31.12.2021.
- Age ≥18y at the time of surgery.
- If possible signed informed consent available.

Exclusion criteria:

- Other types of gastric/esophageal resection, such as transhiatal extended gastrectomy or transthoracic/transcervical subtotal esophagectomy (Ivor-Lewis/McKeown) or not undergoing one of the 4 above specified procedures.
- Emergency (in contrast to elective) gastrectomy.
- No histologically proven gastric adenocarcinoma or other malignancy such as gastrointestinal stromal tumors (GIST).
- Undergoing gastrectomy for a benign indication.
- Age <18y at time of surgery.
- Siewert Type I esophagogastric junction cancer.
- Pregnancy at time of operation.

**Appendix 2: Detailed benchmark in-/exclusion criteria**

Benchmark inclusion criteria:

- **Elective total, distal/subtotal, open or minimally invasive gastrectomy (laparoscopically, robotically assisted)**
  - All above mentioned reconstruction techniques allowed.
- **Histologically proven, primary, resectable (pT1-4a, N0-3b, M0) gastric adenocarcinoma**
  - Exclusion: Tumor stage: pT4b, pM1
- At least **D1+, D2 or D2+ Lymphadenectomy** (LAD)
  - Exclusion: D1 or D3 LAD
- Adult patients of **18-65 years**
  - Exclusion: Age >65years
- **Eastern Cooperative Oncology Group (ECOG) performance status ≤ 1**
  - Exclusion: ECOG Score > 1
- **ASA Score ≤ 2**
  - Exclusion: ASA Score > 2
- **BMI ≤ 30 kg/m2 and >18** at the time of surgery
  - Exclusion: BMI ≤18 or > 30 kg/m2
- Low risk profile with no major comorbidity (please see “exclusion criteria” below)
- Documented follow-up of at least 90 days

Benchmark exclusion criteria:

- **Proximal or pylorus-preserving gastrectomy**
- **Resection of other, adjacent organs**, e.g. pancreatic tail or spleen or multivisceral resection or **administration of** **HIPEC**
  - Allowed: Simultaneous cholecystectomy only
- **Surgery for recurrent gastric cancer, palliative surgery** for metastatic/irresectable gastric cancer
- **History of previous laparotomy or previous**
  - **medium** (such as: hemicolectomy, colonic segmental resection, kidney transplantation)

or

**major** (major liver resection/hemihepatectomy, esophagectomy, duodenopancreatectomy, liver transplantation) **abdominal surgery**

- - Allowed: minor laparoscopic procedures such as appendectomy, cholecystectomy or gynecological procedures such as adnexectomy/hysterectomy.
- **Previous benign gastric surgery**, e.g. bypass/bariatric procedure, surgery for GERD (fundoplication) or vagotomy
- **Previous endoscopic therapy** (e.g. EMR, ESD) for gastric cancer
- **Relevant major comorbidities**, with these being defined as:
  - Cardiovascular: Coronary artery disease, prior myocardial infarction, persistent cardiac arrythmia (e.g. atrial fibrillation), valvular disease, heart failure, peripheral vascular disease, arterial hypertension with complications.
    - Allowed: Hypertension without complication at time of surgery.
  - Respiratory: Chronic obstructive pulmonary disease (FEV1/FVC<0.7), asthma, pulmonary vascular disease (e.g. pulmonary hypertension), obstructive sleep apnea (recurrent episodes of upper airway collapse during sleep, at the time of surgery).
  - Gastrointestinal: Prior gastroduodenal ulcer, persistent GERD, liver cirrhosis, inflammatory bowel disease (ulcerative colitis, Crohn’s disease)
  - Renal: Chronic kidney disease (eGFR < 30ml/min/1.72 m2), hemodialysis
  - Neurological: Cerebrovascular disease, prior stroke or cerebrovascular insult, hemiplegia, dementia, Parkinson’s disease, epilepsy
  - Psychiatric: Depression, schizophrenia
  - Hematological: Anemia, history of thromboembolic events and/or therapeutic anticoagulation.
  - Immunological: Immunosuppression (e.g. due to medication such as steroids, calcineurin inhibitors, etc.), prior organ transplantation (solid & bone marrow), HIV/AIDS, vasculitis/rheumatological diseases
  - Endocrinological: Diabetes mellitus (Type I and Type II, as defined by the American Diabetes Association at the time of surgery), hyperthyroidism, hypothyroid
  - Oncological: Neurological cancer, pulmonary cancer, gastrointestinal cancer, urogenital cancer (incl. Mammary), leukemia, lymphoma, other malignancies

**Appendix 3: Participating centers**

Asia:

- Republic of Korea, Seoul, National University Cancer Hospital
- Japan, Tokyo, Cancer Institute Hospital of the JFCR

Europe:

- France, Nimes, Hôpital Universitaire Carémeau
- Germany, Heidelberg, University Hospital
- Germany, Leipzig, University Hospital
- Germany, Mainz, University Hospital
- Italy, Milano, Niguarda Hospital
- Lithuania, Kaunas, Hospital of Lithuanian University of Health Sciences
- Netherlands, Rotterdam, Erasmus Medical Center
- Spain, Barcelona, Hospital del Mar
- Switzerland, Zurich, University Hospital
- United Kingdom, Birmingham, Queen Elizabeth University Hospital
- United Kingdom, Portsmouth, Queen Alexandra Hospital
- United Kingdom, London, The Royal Marsdon (Prof. A. Chaudry)

South America:

- Brazil, São Paulo, Santa Casa
- Chile, Santiago, Hospital San Borja Arriarán
- Chile, Santiago, Hospital Dr Sotero del Rio, Pontificia Universidad Catolica de Chile

North America:

- USA, Los Angeles/CA, Keck Medical School

Africa:

- South Africa, Johannesburg, Charlotte Maxeke Johannesburg Academic Hospital/ University of the Witwatersrand

GASTRODATA Collaborative (Europe):

- France, Lille, Centre hospitaire universitaire
- Germany, Frankfurt, Agaplesion Markus Hospital
- Germany, Munich, Technical University
- Ireland, Cork, Mercy University Hospital
- Ireland, Dublin, St. James’s Hospital, Trinity College
- Italy, Brescia, University Hospital
- Italy, Forlì, GB Morgagni-L Pierantoni Hospital
- Italy, Milano, Istituto Europeo di Oncologia
- Italy, Milano, San Raffaele Hospital
- Italy, Roma, Cattolica University
- Italy, Torino, University of Torino
- Italy, Verona, University Hospital
- Netherlands, Amsterdam, Academic Medical Center
- Netherlands, Amsterdam, The Netherlands Cancer Institute
- Netherlands, Leiden, University Medical Center
- Poland, Lublin, Medical University
- Poland, Kraków, Jagiellonian University
- Poland, Wroclaw, Medical University
- Portugal, Lisbon, University of Lisbon
- Portugal, Porto, Portuguese Institute of Oncology
- Switzerland, Geneva, University Hospital
- Switzerland, Zurich, Hirslanden Medical Center
- United Kingdom, London, St. Thomas’ NHS
- United Kingdom, London, The Royal Marsden (Prof. W. Allum)
